# Cognitive load scale for AI-assisted L2 writing: scale development and validation

**DOI:** 10.3389/fpsyg.2025.1666974

**Published:** 2025-10-30

**Authors:** Guangyuan Yao, Lingxi Fan

**Affiliations:** ^1^Department of English, University of Macau, Taipa, Macao SAR, China; ^2^Hangzhou Innovation Institute, Beihang University, Hangzhou, China; ^3^Department of Language Science and Technology, The Hong Kong Polytechnic University, Hong Kong, Hong Kong SAR, China

**Keywords:** AI-assisted writing, cognitive load, second language writing, scale development, generative AI, human-AI interaction

## Abstract

This study developed and validated the Cognitive Load Scale for AI-assisted L2 Writing (CL-AI-L2W), an instrument designed to measure the unique cognitive demands of human-AI collaborative writing. As generative AI becomes integral to second language (L2) composition, understanding its impact on cognitive processes is critical. Using a mixed-methods approach grounded in cognitive writing theory and human-AI interaction research, an initial item pool was refined through expert feedback and interviews. An Exploratory Factor Analysis (*N* = 241) on a 35-item draft scale revealed a four-factor structure. A subsequent Confirmatory Factor Analysis (*N* = 305) confirmed this structure with excellent model fit. The final 18-item scale measures four distinct dimensions of cognitive load: (1) Prompt Management, (2) Critical Evaluation, (3) Integrative Synthesis, and (4) Authorial Core Processing. The scale demonstrated excellent internal consistency and strong criterion-related validity through significant correlations with writing anxiety, self-efficacy, and perceived mental effort. As the first validated instrument of its kind, the CL-AI-L2W offers a crucial tool for advancing writing theory and informing pedagogy in AI-enhanced learning environments.

## 1 Introduction

Writing in a second language (L2) is an unequivocally complex and cognitively demanding endeavor (Granena, 2023; Lee, 2005; Zabihi, 2018). It requires the simultaneous orchestration of multiple processes, from high-level planning and idea generation to low-level linguistic encoding and transcription (Hayes, 1996; Kellogg, 1996). The inherent difficulty of this task is compounded by a range of cognitive and affective individual differences that mediate performance. Research has consistently shown that cognitive factors, particularly working memory (WM) capacity, play a significant role in a learner’s ability to manage the intricate demands of L2 composition (Baoshu and Chuanbi, 2015; Baoshu and Luo, 2012; Bergsleithner, 2010; Granena, 2023; Kormos, 2023; Manchón et al., 2023; Zabihi, 2018). Concurrently, affective factors such as writing self-efficacy (Pajares and Valiante, 2006), writing anxiety (Cheng, 2004), and enjoyment (Li et al., 2024) are powerful predictors of writing processes and outcomes. As Flower and Hayes (1980) famously metaphorized, the writer is a busy switchboard operator juggling numerous constraints, a challenge that is amplified in an L2 context where linguistic processes are less automatized (Weigle, 2005; Zabihi, 2018).

The landscape of L2 writing is currently undergoing a paradigm shift with the advent and widespread adoption of generative artificial intelligence (AI) tools like ChatGPT and Bing Chat (Liu et al., 2024). These technologies are not mere aids for proofreading; they are active partners in the composing process, capable of generating ideas, structuring outlines, drafting text, and creating multimodal content (Barrot, 2023; Liu et al., 2024; Su et al., 2023). This integration fundamentally alters the cognitive ecosystem of writing. The cognitive load, traditionally associated with internal processes of planning, translating, and reviewing (Flower and Hayes, 1981; Kellogg, 1996), is now shared, supplemented, and reshaped by the cognitive demands of human-AI interaction. As Liu et al. (2024) demonstrate, new cognitive processes—such as prompt engineering, critical output evaluation, and the synthesis of AI-generated text—have become central to the writing experience.

While existing research has developed instruments to measure the cognitive load of traditional argumentative writing (e.g., Li and Wang, 2024), these scales do not account for the unique cognitive demands imposed by interacting with generative AI. There is a pressing need for a validated measurement tool that can capture this new, hybrid cognitive experience. Understanding the distribution of cognitive load across both traditional writing sub-processes and novel AI-interaction processes is crucial for researchers seeking to model this new form of writing and for educators aiming to develop effective pedagogies for AI-assisted writing (Deng et al., 2023).

The integration of AI tools fundamentally alters the cognitive load profile of L2 writing. According to Cognitive Load Theory (CLT) (Sweller et al., 1998), effective learning occurs when cognitive resources are optimally managed. While AI has the potential to reduce the intrinsic cognitive load associated with linguistic production, it may also introduce new forms of extraneous and germane cognitive load related to human-AI interaction. Understanding this new cognitive architecture is essential for developing effective pedagogy. However, a validated instrument to measure these distinct facets of cognitive load in AI-assisted writing is currently lacking. Therefore, the present study aims to address this critical gap by developing and validating the Cognitive Load Scale for AI-assisted L2 Writing (CL-AI-L2W). Following the rigorous methodological precedents for scale development in the field (e.g., Cheng, 2004; Li and Wang, 2024), this study employs a mixed-methods approach to generate items, establish a robust factor structure, and ensure the scale is a reliable and valid instrument for future research and pedagogical application.

## 2 Literature review

### 2.1 Cognitive demands and processes in writing

Writing in a second language (L2) is a profoundly complex cognitive task that imposes significant demands on a learner’s limited mental resources (Granena, 2023; Kormos, 2023). Foundational cognitive models conceptualize writing as a non-linear, problem-solving activity comprising recursive processes of planning (generating ideas), translating (converting ideas into text), and reviewing (evaluating and revising) (Flower and Hayes, 1981; Hayes, 1996). The successful orchestration of these processes relies heavily on working memory (WM), a limited-capacity system responsible for the temporary storage and manipulation of information (Baddeley and Hitch, 1974). As Kellogg’s (1996) model specifies, the central executive component of WM must coordinate attentional resources to manage content, structure, and audience considerations simultaneously, making writing one of the most demanding tasks for WM (McCutchen, 2000; Olive, 2004).

This cognitive burden is intensified in an L2 context. L2 learners’ linguistic processes, such as lexical retrieval and grammatical encoding, are often less automatized and more effortful (Bereiter, 1980; Scardamalia, 1981; Zimmermann, 2000). Consequently, a substantial portion of their limited WM capacity is consumed by lower-level concerns, leaving fewer resources for higher-level processes like argumentation and organization (Kellogg, 2001; Weigle, 2005; Zabihi, 2018). This phenomenon is effectively explained by Cognitive Load Theory (CLT), which provides a framework for understanding how WM limitations affect learning and performance (Sweller, 1988; Sweller et al., 1998).

CLT distinguishes between three types of cognitive load. Intrinsic cognitive load is the inherent difficulty determined by the complexity of the writing task itself—the number of interacting elements a writer must process simultaneously, such as developing a thesis, organizing paragraphs, and selecting appropriate vocabulary (Sweller, 2010; Sweller et al., 2019). Extraneous cognitive load is generated by suboptimal instructional design or task conditions that consume mental resources without contributing to learning, such as unclear prompts or distracting interfaces (Paas et al., 2003). Finally, germane cognitive load refers to the effortful mental work involved in processing information and constructing long-term schemas, which is essential for developing writing skills (Sweller, 2011). In L2 writing, the high intrinsic load of the task can easily lead to cognitive overload, a state where the total cognitive demand exceeds the capacity of WM (Jiang and Kalyuga, 2022).

While WM capacity and cognitive load are central, a constellation of individual differences mediates their effects on writing performance (Kormos, 2012, 2023). Affective factors, in particular, play a crucial role (McLeod, 1987). Writing anxiety, a skill-specific apprehension, has been consistently linked to poorer performance (Cheng, 2004; Cheng et al., 1999; Choi, 2014; Daly and Miller, 1975; Faigley et al., 1981; Zabihi, 2018), as it may consume WM resources with intrusive thoughts and worries. Conversely, writing self-efficacy—one’s belief in their ability to write successfully—is a robust positive predictor of effort, persistence, and outcomes (Bandura, 1997; Klassen, 2003; Pajares, 2003; Pajares and Valiante, 2006; Prat-Sala and Redford, 2012; Schunk, 2003). These factors are intertwined, with self-efficacy often mediating the negative effects of anxiety (Pajares and Johnson, 1994; Zabihi, 2018). Understanding this interplay between cognitive and affective factors is essential for creating a complete picture of the L2 writing experience.

### 2.2 Measuring cognitive load in writing

Given its theoretical importance, accurately measuring the cognitive load experienced during writing is a key methodological challenge. Early approaches often relied on unidimensional, subjective self-report scales, such as Paas’s (1992) single-item, 9-point scale measuring perceived mental effort. While useful for gauging overall task difficulty (Révész et al., 2016; Robinson, 2001), such measures are insufficient for diagnosing the specific sources of cognitive strain. They cannot distinguish, for example, whether a writer’s high cognitive load stems from difficulties with planning, linguistic expression, or revision (Nückles et al., 2020).

Recognizing this limitation, recent research has moved toward developing multidimensional instruments. The work of Li and Wang (2024) in developing the EFL Argumentative Writing Cognitive Load Scale (EFL-AWCLS) represents a significant advancement and a methodological blueprint for the present study. By grounding their item generation in both cognitive writing theory (Hayes, 2012; Kellogg, 1996) and qualitative data from learners, they developed a reliable and valid scale that captures distinct dimensions of cognitive load, such as argumentation, organization, and language expression. Their work demonstrates the necessity and feasibility of creating a nuanced, multidimensional tool to pinpoint where learners allocate their cognitive resources during traditional writing tasks. Other research has similarly applied a cognitive load perspective to understand the effects of instructional support, such as scaffolding with graphic organizers (Lee and Tan, 2010) or collaborative writing tasks (Jiang and Kalyuga, 2022), further validating CLT as a powerful framework for writing research.

### 2.3 The reconfiguration of cognitive load in AI-assisted writing

The models and measurement tools discussed above were developed for a pre-AI writing environment. The recent integration of generative AI tools like ChatGPT fundamentally reconfigures the cognitive processes and the distribution of cognitive load in writing (Liu et al., 2024). These tools are not passive aids; they are active partners that can generate ideas, draft text, and structure arguments, creating a new, hybrid cognitive ecosystem where cognitive responsibilities are distributed between the human writer and the AI system (Zhao, 2022).

This partnership introduces entirely new, cognitively demanding activities into the writing process. Based on their insightful qualitative investigation, Liu et al. (2024) identified several novel sources of cognitive load:

Prompt management: The writer’s task shifts from solely generating ideas to crafting, refining, and iterating effective prompts to guide the AI. This iterative, metacognitive process represents a significant investment of mental effort.

Critical Evaluation: The writer must serve as a critical gatekeeper of AI-generated content. This involves a heavy cognitive load related to fact-checking for AI “hallucinations” (Lund et al., 2023), identifying potential bias (Lucy and Bamman, 2021), and assessing the relevance and stylistic appropriateness of the output.

Integrative synthesis: The writer must blend AI-generated text with their own. This requires substantial effort to paraphrase for academic integrity (Cotton et al., 2023), maintain a coherent authorial voice, and logically connect disparate pieces of information.

These new processes interact with traditional ones in a complex reallocation of cognitive resources. From a CLT perspective (Sweller, 2011; Sweller et al., 2019), AI can potentially reduce intrinsic load by handling complex sentence construction, but it can also introduce significant extraneous load if its output is inaccurate or irrelevant, forcing the writer to expend effort on evaluation and correction. The effort spent on learning how to prompt the AI effectively and synthesize its output can be seen as a form of germane load—a productive investment in building new human-AI collaboration skills.

### 2.4 The present study

The preceding review highlights a significant and pressing gap: the absence of a psychometrically validated instrument designed to measure the multifaceted cognitive load inherent in the AI-assisted L2 writing process. While qualitative work has provided rich initial insights into the novel cognitive activities involved (Liu et al., 2024), the field lacks a quantitative tool to systematically examine the distribution, antecedents, and consequences of this reconfigured cognitive load. The development of a dedicated, multidimensional scale is a critical next step to advance both cognitive writing theory and evidence-based pedagogy in the age of AI.

Accordingly, this study undertakes the development and validation of the Cognitive Load Scale for AI-assisted L2 Writing (CL-AI-L2W). To guide this process, the study is structured around the following research questions:

RQ1: What are the underlying dimensions (or factors) of cognitive load experienced by second language (L2) learners during an AI-assisted writing task?
RQ2: To what extent is the newly developed Cognitive Load Scale for AI-assisted L2 Writing (CL-AI-L2W) a reliable and valid instrument for measuring this construct?

## 3 Method

This study employed a sequential mixed-methods research design to develop and validate the Cognitive Load Scale for AI-assisted L2 Writing (CL-AI-L2W). The research was conducted in three major phases, following established best practices in scale development (DeVellis, 2017; Li and Wang, 2024): (1) Item generation and content validity assessment, (2) A pilot study for item refinement and Exploratory Factor Analysis (EFA), and (3) A main study for Confirmatory Factor Analysis (CFA) and further validity and reliability testing.

### 3.1 Item generation and content validity

The initial item pool was generated through a two-pronged approach to ensure both theoretical grounding and ecological validity.

First, the item development process was guided by a clear theoretical framework to ensure comprehensive coverage of the construct. Drawing from the literature review, we identified five *a priori* theoretical domains expected to constitute the cognitive load in AI-assisted L2 writing. Two domains represented traditional writing processes, informed by the models of Flower and Hayes (1981), Kellogg (1996), and the dimensions of the EFL-AWCLS (Li and Wang, 2024). Three domains represented novel AI-interaction processes, derived from the qualitative findings of Liu et al. (2024).

Based on this five-domain framework (Table 1), we generated an initial pool of 48 items. Items for the traditional domains were adapted from existing literature and the EFL-AWCLS, while items for the AI-interaction domains were developed based on the specific cognitive activities described by Liu et al. (2024). To ensure the items were grounded in learners’ authentic experiences, we conducted semi-structured interviews with a small, purposive sample of 12 L2 learners (intermediate to advanced proficiency) who had experience using generative AI for writing. Participants were asked to complete a short AI-assisted writing task and then describe the mental effort they invested in different parts of the process. The language and concepts they used were incorporated into the item wording.

**Table 1:** Five-domain framework for the initial pool of items.

Domain number	Domain name	Core focus	Category
1	Planning and organization	Foundational stages of writing: outlining, structuring, and organizing ideas.	Traditional writing processes
2	Language expression and revision	Crafting and refining text for clarity, style, and grammatical correctness.	Traditional writing processes
3	Prompt engineering and management	Formulating, iterating, and managing instructions for AI tools.	AI interaction
4	Critical output evaluation	Critically assessing AI-generated content for accuracy, bias, and relevance.	Ai interaction
5	Integration and synthesis	Combining AI-generated content with original writing to create a cohesive final product.	AI interaction and writing

This process resulted in an initial pool of 48 items, each framed as a statement about the mental effort required for a specific activity (e.g., “How much mental effort did it take you to design effective prompts for the AI?”). All items were measured on a 7-point Likert scale, ranging from 1 (very, very low mental effort) to 7 (very, very high mental effort), consistent with established cognitive load measurement (Paas, 1992).

The initial 48-item pool was submitted to a panel of six experts for content validity assessment. The panel comprised three associate professors specializing in L2 writing and psycholinguistics, and three doctoral candidates whose research focuses on AI in language education. The experts were asked to evaluate each item based on its relevance (is it relevant to the construct of AI-assisted writing cognitive load?) and clarity (is the wording unambiguous?). They provided both quantitative ratings and qualitative feedback. Items that received low ratings for relevance or were flagged as unclear by more than two experts were revised or eliminated. This process reduced the item pool to a refined set of 35 items for the pilot study.

### 3.2 Pilot study and exploratory factor analysis (EFA)

A total of 258 L2 learners were recruited from a university in China to participate in the pilot study. After removing incomplete responses, the final sample for EFA consisted of 241 participants (155 female, 86 male). Their ages ranged from 18 to 23 (M = 20.1, SD = 1.5). All were non-English majors who had passed the CET-4 or CET-6, indicating an intermediate-to-high intermediate English proficiency level. All participants reported having used generative AI tools (e.g., ChatGPT, Bing Chat, Deepseek) for academic tasks prior to the study. Data was collected online. Participants were first given a standardized set of instructions and a 10-min tutorial on using Deepseek for an argumentative writing task. They were then presented with an argumentative writing prompt (“Should universities invest more in AI-based educational tools?”) and given 40 min to write a 250-word essay using Deepseek as an assistant. Interactions with the AI tool were conducted on the public DeepSeek V3.1 interface (accessed in https://chat.deepseek.com/). Participants were explicitly instructed not to enter any personal or identifiable information into the AI chat prompt. To further protect privacy, the AI chat logs were not collected or logged by the research team. The study’s data collection was limited to the final written essays produced by the participants and their questionnaire responses. This approach ensured that no direct interactions with the AI system were stored, safeguarding participant privacy.

Immediately after completing the task, they were directed to a questionnaire containing the 35-item draft scale. Prior to the main analyses, the data from both samples were screened for accuracy, missing values, outliers, and assumptions of normality. Data entry accuracy was verified through a random check of 10% of the cases. The rate of missing data was minimal (<1%) and handled using pairwise deletion, which is appropriate for a low volume of missingness. Multivariate outliers were assessed using Mahalanobis distance (*p* < 0.001); no cases were identified as significant outliers requiring removal. Finally, the assumptions of univariate normality were checked by examining skewness and kurtosis values for all 18 final scale items. All values fell within the acceptable range of −2 to +2, indicating that the data did not significantly deviate from a normal distribution.

To address RQ1, an Exploratory Factor Analysis (EFA) was conducted using SPSS (Version 28). First, item analysis was performed, and items with corrected item-total correlations below 0.40 were removed. The suitability of the data for factor analysis was confirmed using the Kaiser-Meyer-Olkin (KMO) measure and Bartlett’s Test of Sphericity.

A Principal Axis Factoring (PAF) with Oblimin rotation was chosen, as the underlying factors of cognitive load were theoretically expected to be correlated. The number of factors to retain was determined by multiple criteria: (a) parallel analysis, (b) the Kaiser criterion (eigenvalues > 1), (c) examination of the scree plot, and (d) the theoretical interpretability of the resulting factors. The parallel analysis, which is considered the most robust method, clearly suggested a four-factor solution. Items were retained if their primary factor loading was above 0.40 and they did not exhibit significant cross-loadings (i.e., a loading > 0.30 on a secondary factor).

### 3.3 Main study and confirmatory factor analysis (CFA)

A second, independent sample of 312 L2 learners was recruited from a different university to avoid sample overlap. After data screening, the final sample for the main study comprised 305 participants (198 female, 107 male), with a similar demographic profile to Sample 1 (Age: M = 20.5, SD = 1.7; intermediate-to-high intermediate English proficiency).

In addition to the refined CL-AI-L2W scale derived from the EFA, the following established instruments were administered to assess criterion-related validity:

Subjective Mental Effort Scale: A single-item, 9-point scale adapted from Paas (1992) to measure overall perceived task difficulty, used for convergent validity.

Second Language Writing Anxiety Inventory (SLWAI): The 22-item scale developed by Cheng (2004) to measure writing apprehension.

Writing Self-Efficacy Scale: A 10-item subscale adapted from Pajares and Valiante (2006) measuring learners’ confidence in their ability to perform writing tasks.

The procedure for the main study was identical to that of the pilot study. After completing the AI-assisted argumentative writing task, participants were directed to a questionnaire booklet. This booklet always began with the final 18-item CL-AI-L2W scale, followed by the three validation scales (Second Language Writing Anxiety Inventory, Writing Self-Efficacy Scale, and the single-item mental effort scale). To minimize potential order effects among the validation scales, their presentation order was counterbalanced across participants using a Latin square design. Specifically, six possible orderings of the three validation scales were created, and participants were randomly assigned to one of these six versions of the questionnaire booklet.

To address RQ2, a comprehensive set of analyses was conducted to establish the reliability and validity of the CL-AI-L2W. All analyses, unless otherwise specified, were performed using SPSS (Version 28) and Mplus (Version 8.8). First, a Confirmatory Factor Analysis (CFA) was performed on the data from Sample 2 to test the four-factor structure identified in the EFA. Given the 7-point ordinal nature of the Likert-scale items, we employed the Weighted Least Squares Mean and Variance Adjusted (WLSMV) estimator, which is robust for such data. Model fit was evaluated against established criteria: χ^2^/df < 3, CFI > 0.95, TLI > 0.95, RMSEA < 0.06, and WRMR < 1.0 (Hu and Bentler, 1999; Yu, 2002). Second, we assessed construct validity in detail. Convergent validity was examined by calculating the Average Variance Extracted (AVE), and discriminant validity was tested using the Fornell-Larcker criterion and the Heterotrait-Monotrait Ratio of Correlations (HTMT). Third, internal consistency reliability was assessed using both Cronbach’s alpha and McDonald’s omega (*ω*) coefficients, with values above 0.70 considered acceptable. Measurement invariance of the scale was also tested across gender and the two study samples (EFA vs. CFA) to ensure its psychometric equivalence across groups. Finally, criterion-related validity was examined through Pearson correlation analyses, investigating the relationships between the CL-AI-L2W scores and the scores from the other validated scales (overall mental effort, writing anxiety, and writing self-efficacy). It was hypothesized that the CL-AI-L2W would show a strong positive correlation with mental effort, a moderate positive correlation with writing anxiety, and a moderate negative correlation with writing self-efficacy (see Table 2).

**Table 2:** *A priori* item blueprint and distribution across stages.

Domain	Definition (summary)	Initial pool (48)	Post–expert review (35)	Final validated scale (18)
Planning and organization (PO)	Deciding argument, outlining, structuring	10	7	2 (merged into Authorial Core Processing)
Prompt management (PM)	Designing and refining prompts for AI	8	6	5
Critical evaluation (CE)	Evaluating AI outputs for accuracy, relevance, bias, style	9	7	5
Integration and synthesis (IS)	Paraphrasing and blending AI with own text	8	6	4
Language expression and revision (LER)	Vocabulary, grammar, coherence, revision	13	9	2 (merged into Authorial Core Processing)
Total	–	48	35	18

## 4 Results

### 4.1 Exploratory factor analysis

To answer RQ1, an EFA was performed on the data from the pilot study (Sample 1, *N* = 241) to identify the underlying dimensions of cognitive load in AI-assisted L2 writing.

First, item analysis was conducted on the initial 35 items. Five items were removed due to low corrected item-total correlations (< 0.40). These items, along with their correlation values, were: Item 34 (“Concentrate on the writing task without getting distracted”): r = 0.31; Item 33 (“Manage your time effectively”): r = 0.34; Item 29 (“Focus on spelling and punctuation”): r = 0.35; Item 5 (“Ensure a smooth and logical flow between paragraphs”): r = 0.38; Item 13 (“Manage the conversation with the AI”): r = 0.39.

The remaining 30 items were subjected to Principal Axis Factoring (PAF). The suitability of the data for factor analysis was confirmed, with a high Kaiser-Meyer-Olkin (KMO) value of 0.92 and a significant Bartlett’s Test of Sphericity (χ^2^(435) = 3854.21, *p* < 0.001). The PAF with Oblimin rotation initially yielded five factors with eigenvalues greater than 1. However, the scree plot clearly showed an elbow after the fourth factor, and the fifth factor was weak and difficult to interpret. Therefore, a four-factor solution was specified, which was theoretically more coherent and parsimonious. During this process, a further 12 items were removed because they either had primary factor loadings below 0.40 or exhibited significant cross-loadings (> 0.32) on more than one factor.

The final EFA resulted in a clean and interpretable four-factor structure comprising 18 items, which collectively explained 71.84% of the total variance. The factor loadings for each subscale are presented in Table 3. All items loaded strongly on their respective factors (ranging from 0.69 to 0.87), and all subscales demonstrated excellent internal consistency (*α* ≥ 0.85).

**Table 3:** EFA results and item status.

Item no.	Item content summary	Factor loadings (F1, F2, F3, F4)	Status and rationale for decision
10	Rephrase/refine prompts when AI’s answer was not helpful.	0.87, 0.11, 0.09, 0.03	Retained: strong, clean loading on F1 (PM)
8	Figure out the best way to phrase initial questions.	0.85, 0.15, 0.12, 0.08	Retained: strong, clean loading on F1 (PM)
12	Ask effective follow-up questions.	0.81, 0.18, 0.10, 0.05	Retained: strong, clean loading on F1 (PM)
9	Think of specific keywords to guide the AI.	0.79, 0.13, 0.08, 0.11	Retained: strong, clean loading on F1 (PM)
11	Break down a complex task into smaller prompts.	0.75, 0.09, 0.16, 0.14	Retained: strong, clean loading on F1 (PM)
15	Evaluate if AI suggestions were relevant to your argument.	0.12, 0.86, 0.14, 0.07	Retained: Strong, clean loading on F2 (CE)
17	Decide which parts of AI output to use and which to ignore.	0.10, 0.83, 0.21, 0.10	Retained: Strong, clean loading on F2 (CE)
14	Judge if AI information was factually accurate.	0.08, 0.81, 0.11, 0.05	Retained: Strong, clean loading on F2 (CE)
16	Assess the tone and style of the AI text.	0.14, 0.77, 0.19, 0.13	Retained: Strong, clean loading on F2 (CE)
18	Check the AI text for potential bias.	0.06, 0.72, 0.09, 0.08	Retained: Strong, clean loading on F2 (CE)
22	Blend AI text smoothly with your own writing.	0.11, 0.18, 0.85, 0.15	Retained: Strong, clean loading on F3 (IS)
21	Paraphrase or rewrite AI sentences in your own words.	0.09, 0.15, 0.82, 0.12	Retained: Strong, clean loading on F3 (IS)
24	Connect your own ideas logically with AI ideas.	0.13, 0.20, 0.80, 0.19	Retained: Strong, clean loading on F3 (IS)
23	Ensure your personal authorial voice was not lost.	0.07, 0.12, 0.74, 0.25	Retained: Strong, clean loading on F3 (IS)
3	Create a logical structure or outline for the essay.	0.09, 0.10, 0.17, 0.84	Retained: Strong, clean loading on F4 (ACP)
1	Decide on the main argument or position for your essay.	0.11, 0.08, 0.11, 0.80	Retained: Strong, clean loading on F4 (ACP)
28	Construct grammatically correct English sentences.	0.05, 0.06, 0.14, 0.73	Retained: Strong, clean loading on F4 (ACP)
27	Find the right vocabulary to express your ideas precisely.	0.08, 0.10, 0.12, 0.69	Retained: Strong, clean loading on F4 (ACP)
20	Identify awkward phrasing in AI text.	0.15, 0.51, 0.28, 0.43	Removed (EFA): Significant cross-loading on F2 (CE) and F4 (ACP)
30	Revise sentences you wrote yourself.	0.08, 0.13, 0.44, 0.49	Removed (EFA): Significant cross-loading on F3 (IS) and F4 (ACP)
31	Review entire essay for overall coherence.	0.18, 0.29, 0.39, 0.46	Removed (EFA): Significant cross-loading and primary loading is weak (<0.50)
4	Organize arguments within each paragraph.	0.06, 0.11, 0.25, 0.42	Removed (EFA): Conceptually redundant with Item 3; weaker loading
25	Maintain consistent flow between your text and AI’s.	0.10, 0.22, 0.53, 0.28	Removed (EFA): Redundant with Item 22; weaker loading
32	Ensure final text answered the prompt.	0.21, 0.41, 0.28, 0.25	Removed (EFA): Redundant with Item 15; weaker loading
35	Monitor overall logic of your argument.	0.19, 0.26, 0.23, 0.48	Removed (EFA): Redundant with Item 1; weaker loading
7	Think about the intro and conclusion.	0.15, 0.18, 0.30, 0.38	Removed (EFA): Primary loading < 0.40
6	Decide what info you needed to find/generate.	0.28, 0.25, 0.15, 0.37	Removed (EFA): Primary loading < 0.40
2	Come up with initial ideas on your own.	0.12, 0.09, 0.21, 0.35	Removed (EFA): Primary loading < 0.40
19	Compare different responses from the AI.	0.25, 0.31, 0.38, 0.19	Removed (EFA): Primary loading < 0.40
26	Synthesize info from multiple AI responses.	0.21, 0.28, 0.39, 0.22	Removed (EFA): Primary loading < 0.40
13	Manage the conversation with the AI.	N/A	Removed (Item Analysis): Corrected item-total correlation < 0.40
5	Ensure a smooth and logical flow between paragraphs.	N/A	Removed (Item Analysis): Corrected item-total correlation < 0.40
29	Focus on spelling and punctuation.	N/A	Removed (Item Analysis): Corrected item-total correlation < 0.40
33	Manage your time effectively.	N/A	Removed (Item Analysis): Corrected item-total correlation < 0.40
34	Concentrate on the writing task without getting distracted.	N/A	Removed (Item Analysis): Corrected item-total correlation < 0.40

Principal Axis Factoring with Oblimin rotation was performed on 30 items. Item numbers correspond to the draft scale in Appendix A. Removal criteria: (1) Corrected item-total correlation < 0.40; (2) EFA primary loading < 0.40 or a cross-loading > 0.32.

### 4.2 Confirmatory factor analysis (CFA)

To further test the four-factor structure of the CL-AI-L2W identified in the EFA (RQ2), a CFA was conducted on the data from the main study (Sample 2, N = 305). Following the methodological recommendations for ordinal data, the model was estimated using the Weighted Least Squares Mean and Variance Adjusted (WLSMV) estimator. The results demonstrated an excellent fit of the hypothesized four-factor model to the data. The goodness-of-fit indices were robust: χ^2^(129) = 265.82, *p* < 0.001; χ^2^/df = 2.06; CFI = 0.97; TLI = 0.96; RMSEA = 0.059 (90% CI = [0.050, 0.068]); and WRMR = 0.95. All these indices met or exceeded the stringent criteria for good model fit (e.g., CFI/TLI > 0.95, RMSEA < 0.06, WRMR < 1.0), providing strong empirical support for the four-factor structure of the scale.

**Figure 1:**
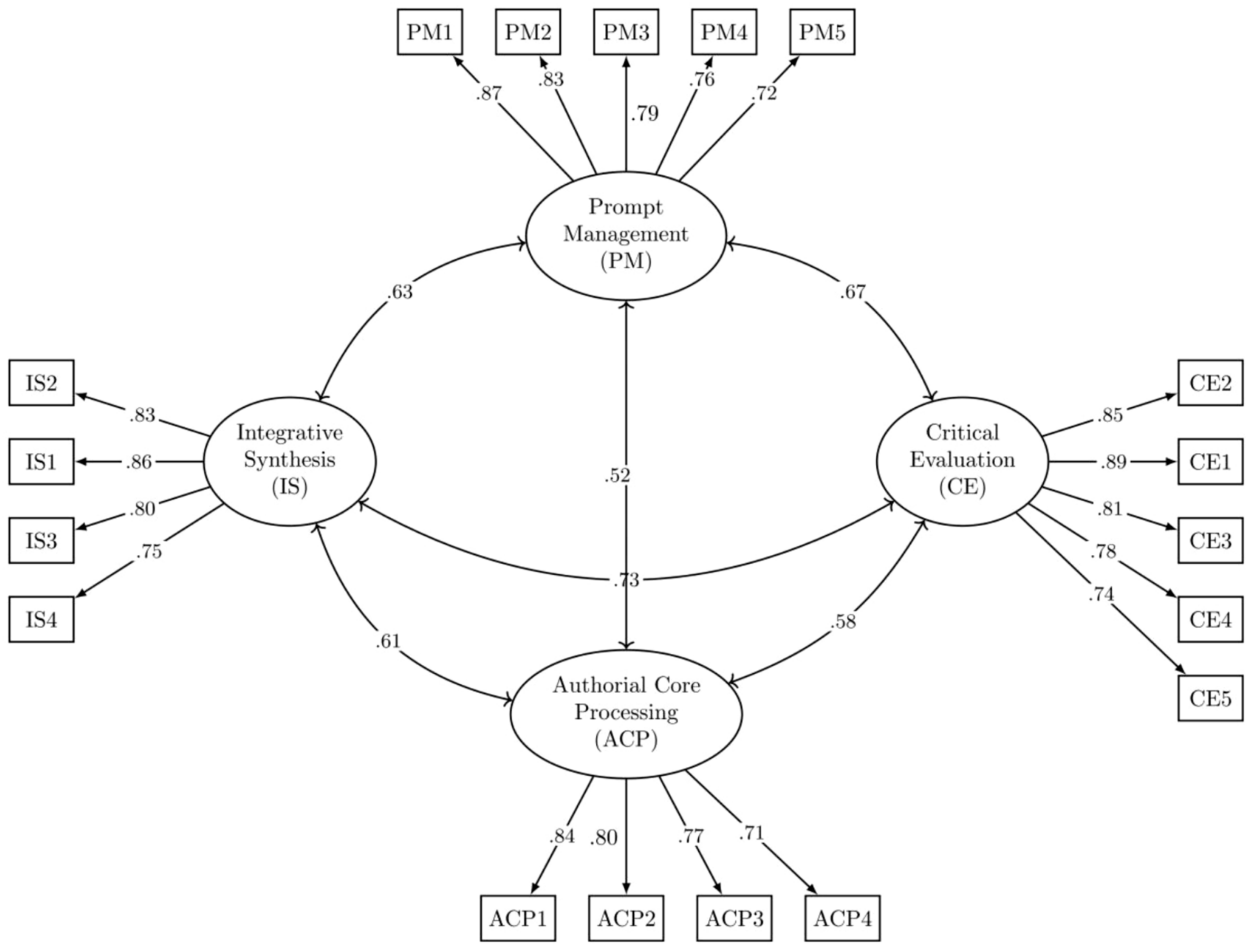
Standardized path diagram of the four-factor CFA model for the CL-AI-L2W.

As shown in the final model (see Figure 1), all standardized factor loadings were statistically significant (*p* < 0.001) and substantial, ranging from 0.71 to 0.89. This indicates that all 18 items are strong and reliable indicators of their respective latent constructs. The correlations between the four latent factors were moderate to strong, ranging from r = 0.52 (between Prompt Management and Authorial Core Processing) to r = 0.73 (between Critical Evaluation and Integrative Synthesis). These correlations confirm that the factors are distinct yet related components of the overarching construct of cognitive load in AI-assisted writing, justifying the use of an oblique rotation in the EFA. The final CFA model is depicted in Figure 1.

### 4.3 Reliability and criterion-related validity

The reliability for the overall 18-item CL-AI-L2W scale was excellent, with a Cronbach’s alpha of 0.94. The subscale reliabilities, as reported in Table 3, were also high (PM: 0.91; CE: 0.89; IS: 0.88; ACP: 0.85).

To establish criterion-related validity, Pearson correlations were calculated between the CL-AI-L2W (total and subscale scores) and the other measures administered in the main study. Descriptive statistics and the correlation matrix are presented in Table 4.

**Table 4:** Descriptive statistics and Pearson correlations among variables.

Variable	M	SD	1	2	3	4	5	6	7
1. CL-AI-L2W total	4.31	1.15	–						
2. Prompt management	4.55	1.30	0.81**	–					
3. Critical evaluation	4.81	1.25	0.85**	0.65**	–				
4. Integrative synthesis	4.40	1.28	0.83**	0.61**	0.71**	–			
5. Authorial core processing	3.48	1.21	0.76**	0.48**	0.55**	0.58**	–		
6. Overall mental effort	6.52	1.45	0.72	0.60**	0.68**	0.64**	0.51**	–	
7. Writing anxiety	3.15	0.98	0.45	0.38**	0.49**	0.41**	0.35**	0.48**	–
8. Writing self-efficacy	3.88	1.05	−0.51	−0.42**	−0.55**	−0.47**	−0.40**	−0.53**	−0.62**

*p < 0.05, **p < 0.01.

As hypothesized, the total CL-AI-L2W score showed a strong, positive correlation with the single-item overall mental effort scale (r = 0.72, *p* < 0.01), supporting its convergent validity. Furthermore, the total score was moderately and positively correlated with writing anxiety (r = 0.45, *p* < 0.01) and moderately and negatively correlated with writing self-efficacy (r = −0.51, *p* < 0.01). These significant correlations provide strong evidence for the criterion-related validity of the new scale. The final 18-item CL-AI-L2W is presented in Appendix B.

A series of analyses were conducted to establish the reliability and validity of the CL-AI-L2W. Table 5 presents a summary of the descriptive statistics, reliability coefficients, and validity assessments. The internal consistency of the subscales was excellent. As shown in Table 5, both Cronbach’s alpha (*α*) and McDonald’s omega (*ω*) coefficients for all four factors were well above the recommended 0.80 threshold, ranging from 0.87 to 0.93. Convergent validity was strongly supported, with the Average Variance Extracted (AVE) for each factor exceeding the 0.50 criterion (ranging from 0.63 to 0.70). This indicates that, on average, more than 63% of the variance in the items was accounted for by their respective latent construct. Furthermore, the strong and significant factor loadings reported in the CFA (Section 4.2) provide additional evidence for convergent validity. We assessed discriminant validity using two rigorous methods. First, following the Fornell-Larcker criterion, the square root of the AVE for each construct was greater than its correlation with any other construct, providing initial support for discriminant validity. Second, we calculated the Heterotrait-Monotrait Ratio of Correlations (HTMT). All HTMT values were well below the conservative threshold of 0.85, ranging from 0.59 (PM-ACP) to 0.81 (CE-IS), offering strong evidence that the four factors are empirically distinct constructs.

**Table 5:** Descriptive statistics, reliability, and validity assessment for the CL-AI-L2W subscales.

Variable	M	SD	ω	α	AVE	1	2	3	4
1. Prompt management	4.55	1.30	0.92	0.91	0.68	0.82			
2. Critical evaluation	4.81	1.25	0.93	0.92	0.70	0.67	0.84		
3. Integrative synthesis	4.40	1.28	0.90	0.89	0.67	0.63	0.73	0.82	
4. Authorial core processing	3.48	1.21	0.88	0.87	0.63	0.52	0.58	0.61	0.79

*N* = 305. M, Mean; SD, Standard Deviation; ω, McDonald’s Omega; α, Cronbach’s Alpha; AVE, Average Variance Extracted. The diagonal elements in bold are the square roots of the AVE. Off-diagonal elements are the latent factor correlations derived from the CFA model. For discriminant validity, diagonal values should be greater than the off-diagonal values in their respective rows and columns.

### 4.4 Measurement invariance

To ensure that the CL-AI-L2W functions equivalently across different subgroups, we conducted multi-group CFA to test for measurement invariance across gender (male vs. female) and the two study samples (EFA sample vs. CFA sample). We tested a sequence of nested models for configural, metric (factor loadings), and scalar (intercepts) invariance. The results are summarized in Table 6. As shown in Table 6, for both gender and sample comparisons, all models demonstrated excellent fit to the data. Crucially, the change in the Comparative Fit Index (ΔCFI) between nested models was consistently minimal. For gender, the ΔCFI was 0.002 for both metric and scalar invariance. For the sample comparison, the ΔCFI was 0.001 for metric and 0.002 for scalar invariance. As all ΔCFI values were well below the recommended cutoff of 0.01, strong evidence for scalar invariance was established. This indicates that the scale’s factor structure, item loadings, and item intercepts are equivalent across these groups, supporting the validity of comparing latent mean scores between genders and across the two samples in future research.

**Table 6:** Measurement invariance testing across gender and sample.

Model	χ^2^	df	CFI	TLI	RMSEA [90% CI]	Δχ^2^	Δdf	ΔCFI
Invariance across gender (male vs. female)								
M1: Configural	485.21	258	0.972	0.966	0.055 [0.048, 0.062]	–	–	–
M2: Metric	499.86	272	0.970	0.965	0.054 [0.047, 0.061]	14.65	14	0.002
M3: Scalar	515.03	286	0.968	0.964	0.053 [0.046, 0.060]	15.17	14	0.002
Invariance across sample (EFA vs. CFA)								
M4: Configural	510.19	258	0.975	0.970	0.045 [0.039, 0.051]	-	-	-
M5: Metric	523.88	272	0.974	0.970	0.044 [0.038, 0.050]	13.69	14	0.001
M6: Scalar	540.25	286	0.972	0.968	0.044 [0.038, 0.050]	16.37	14	0.002

*N* = 546. CFI, Comparative Fit Index; TLI, Tucker-Lewis Index; RMSEA, Root Mean Square Error of Approximation; CI, Confidence Interval. ΔCFI values ≤ 0.01 between nested models support invariance.

## 5 Discussion

The primary purpose of this study was to develop and validate the first psychometrically sound instrument, the Cognitive Load Scale for AI-assisted L2 Writing (CL-AI-L2W), to measure the multifaceted cognitive demands faced by L2 learners in the new era of generative AI. The rigorous, multi-phase methodology yielded an 18-item, four-factor scale with excellent reliability and strong evidence of validity. The findings not only address the critical gap identified in the literature but also provide novel insights into the evolving cognitive architecture of L2 writing.

The first research question sought to identify the underlying dimensions of cognitive load in AI-assisted L2 writing. The Exploratory Factor Analysis, confirmed by the subsequent CFA, revealed a clear and theoretically coherent four-factor structure: (1) Prompt Management, (2) Critical Evaluation, (3) Integrative Synthesis, and (4) Authorial Core Processing. This structure provides the first quantitative evidence for the cognitive reconfiguration of the writing process previously described in qualitative research.

The emergence of Prompt Management, Critical Evaluation, and Integrative Synthesis as distinct and robust factors empirically validates the foundational qualitative work of Liu et al. (2024). While their study identified these new processes descriptively, the present study demonstrates that they represent quantifiable and separate sources of cognitive load. Notably, Critical Evaluation emerged as the dimension with the highest mean score (M = 4.81), suggesting that the most mentally demanding task for L2 learners is not generating text, but acting as a critical gatekeeper for AI-generated content. This involves a heavy cognitive investment in assessing relevance, accuracy, and style, a finding that underscores the importance of developing students’ critical AI literacy (Lund et al., 2023; Walters and Wilder, 2023).

The Integrative Synthesis factor captures the cognitive effort required to blend AI output with one’s own writing, a process that requires maintaining authorial voice and ensuring coherence (Cotton et al., 2023). Together, these three AI-centric factors illustrate a fundamental shift: cognitive load is no longer solely an internal phenomenon related to memory retrieval and sentence generation but is now heavily situated in the interactive, dialogic space between the writer and the AI. From a working memory perspective, these three AI-centric factors appear to impose a heavy burden primarily on the central executive (Baddeley and Hitch, 1974; Kellogg, 1996). Critical Evaluation and Integrative Synthesis, in particular, require the central executive to perform several demanding functions simultaneously: constantly switching attention between the AI’s output and one’s own mental model of the text, inhibiting irrelevant or inaccurate AI suggestions, and continuously updating the writing plan. This constant monitoring and decision-making process is a hallmark of executive control and explains why these factors emerged as significant sources of cognitive load. Prompt Management also taxes the central executive, as it involves goal setting, planning a sequence of queries, and monitoring the effectiveness of the human-AI dialogue.

Perhaps the most revealing finding is the fourth factor, Authorial Core Processing. This dimension combines high-level planning (e.g., deciding on an argument, creating a structure) and core linguistic encoding (e.g., grammar, lexis), which are central to traditional writing models (Kellogg, 1996; Li and Wang, 2024). The fact that these traditional elements clustered together into a single, distinct factor suggests that even with AI assistance, a fundamental core of authorial responsibility remains. However, the relatively lower mean score for this factor (M = 3.48) compared to the AI-interaction factors is highly significant. It provides empirical support for the hypothesis that AI tools offload a substantial portion of the cognitive burden traditionally associated with planning and translating (Flower and Hayes, 1981), thereby freeing up cognitive resources that are immediately reallocated to the new, demanding tasks of prompting, evaluating, and integrating. Interpreted through the lens of working memory, the lower cognitive load on Authorial Core Processing suggests that AI assistance may offload some of the demands typically placed on WM’s subsidiary systems. For instance, AI’s ability to quickly generate grammatically correct sentences and suggest vocabulary could reduce the burden on the phonological loop, which is heavily involved in linguistic encoding (Kellogg, 1996). Similarly, AI’s capacity to help structure an outline might lessen the strain on the visuospatial sketchpad during planning. This offloading appears to free up limited central executive resources, which, as our data show, are then immediately reallocated to the novel and highly demanding tasks of managing the human-AI interaction. This finding empirically illustrates a critical reallocation of cognitive resources within the writer’s working memory system, a shift from internal content generation to external tool management and evaluation.

The second research question concerned the reliability and validity of the new scale. The results provide compelling evidence that the 18-item CL-AI-L2W is a robust and trustworthy instrument. The excellent internal consistency of the overall scale (*α* = 0.94) and its subscales (α ranging from 0.85 to 0.91) indicates that the items within each factor reliably measure a single underlying construct.

The confirmatory factor analysis strongly supported the four-factor model, with all goodness-of-fit indices meeting or exceeding stringent criteria (Hu and Bentler, 1999). This confirms that the structure identified in the EFA is not a statistical artifact of one sample but is a stable representation of the construct. The criterion-related validity analyses further strengthen the case for the scale’s utility. The strong positive correlation with a global measure of mental effort (Paas, 1992) provides convergent validity, showing that the CL-AI-L2W indeed measures cognitive load.

Crucially, the scale behaves as expected within the broader nomological network of writing psychology. The moderate positive correlation with writing anxiety (r = 0.45) aligns with established research showing that cognitively demanding tasks can exacerbate anxiety (Cheng, 2004; Zabihi, 2018). Conversely, the moderate negative correlation with writing self-efficacy (r = −0.51) supports the notion that learners who feel more confident in their abilities perceive the writing task as less mentally burdensome (Pajares and Valiante, 2006). These relationships demonstrate that the CL-AI-L2W is not only measuring cognitive load in isolation but is also meaningfully connected to the key affective factors that mediate the L2 writing experience.

The findings of this study carry significant implications for both theoretical understanding and pedagogical application. From a theoretical perspective, the validated four-factor structure of the CL-AI-L2W invites a reconsideration of existing cognitive models of writing. While classical models such as those of Flower and Hayes (1981) and Kellogg (1996) continue to provide valuable insights into the core processes of authorship, they are increasingly inadequate in capturing the distributed, interactive nature of AI-assisted composition. The results of this study suggest the need to conceptualize AI-assisted writing as a hybrid cognitive ecosystem in which the cognitive load is dynamically distributed between the writer’s internal cognitive resources and the external cognitive affordances provided by AI systems. Within this framework, the CL-AI-L2W serves as a diagnostic tool to empirically map how cognitive responsibilities are allocated and managed during AI-mediated writing.

Pedagogically, the implications of this model are both immediate and actionable. The CL-AI-L2W can function as an effective instrument for diagnosing specific areas of cognitive strain that students encounter in the process of AI-assisted composition. By administering the scale, educators can identify whether learners experience the greatest difficulty in formulating effective prompts, critically evaluating AI-generated content, integrating and synthesizing information, or managing core authorial processes. Such insights enable the design of targeted instructional interventions tailored to students’ specific cognitive challenges. For instance, elevated scores in the dimension of prompt management would indicate the need to strengthen students’ proficiency in formulating clear, effective queries and engaging in productive dialogue with AI systems. Similarly, high scores in critical evaluation underscore the urgency of cultivating students’ digital literacy skills, including the ability to assess the credibility, bias, and stylistic appropriateness of AI-generated outputs. Where integrative synthesis scores are elevated, instructional focus may need to be placed on helping students paraphrase, summarize, and integrate information while maintaining a coherent and authentic authorial voice.

At the same time, the relatively lower cognitive load associated with authorial core processing offers both promise and caution. On one hand, this pattern may reflect the supportive role that AI can play in helping students overcome linguistic barriers, allowing them to allocate more cognitive resources to higher-order thinking and organization. On the other hand, there is a risk that essential skills related to argument construction, critical reasoning, and language production may be underdeveloped or gradually atrophied due to overreliance on AI-generated content. Consequently, while AI tools can enhance certain dimensions of the writing process, educators must remain attentive to the need for preserving and fostering core writing competencies to ensure that learners do not become passive recipients of machine-generated text, but remain active, critical, and creative agents in the composition process.

## 6 Conclusion

The advent of generative AI represents a paradigm shift in L2 writing, fundamentally altering the cognitive demands of the composition process. This study successfully developed and validated the first Cognitive Load Scale for AI-assisted L2 Writing (CL-AI-L2W), an 18-item, four-factor instrument that is both reliable and valid. The scale reveals that the cognitive load in this new environment is a hybrid construct, comprising the novel demands of Prompt Management, Critical Evaluation, and Integrative Synthesis, alongside the enduring demands of Authorial Core Processing. By providing the field with a robust tool to measure this complex construct, this study lays the groundwork for a new generation of research aimed at understanding, modeling, and ultimately improving L2 writing pedagogy in the age of artificial intelligence.

While this study makes a significant contribution, several limitations should be acknowledged. First, the participants were Chinese university students of intermediate-to-high proficiency. Future research should validate the CL-AI-L2W with learners from different L1 backgrounds, proficiency levels, and educational contexts to establish its broader generalizability. Second, the study focused on a single argumentative writing task using one AI system. The distribution of cognitive load is likely to vary across different genres and tasks (e.g., creative writing, summary writing). Finally, for convergent validity, we employed a single-item measure of overall mental effort. While widely used, the psychometric properties of single-item measures, such as their reliability, cannot be assessed with the same rigor as multi-item scales. Future studies could incorporate additional measures or employ a test–retest design to further strengthen the validity evidence.

The development of the CL-AI-L2W opens up numerous avenues for future research. Researchers can now move beyond description to systematic, quantitative investigation. For instance, experimental studies could use the scale as an outcome measure to compare the effectiveness of different AI training interventions. Longitudinal studies could track how the cognitive load profile of learners changes as they gain more expertise in human-AI collaboration. Finally, future studies could correlate the CL-AI-L2W subscale scores with objective measures of the writing process (e.g., keystroke logging, revision patterns) and product (e.g., CAF measures) to build a more comprehensive model of AI-assisted L2 writing.

## Data Availability

The original contributions presented in the study are included in the article/supplementary material, further inquiries can be directed to the corresponding author.
