# Supplementary material for: Cognitive load scale for AI-assisted L2 writing: scale development and validation
Source: Front Psychol. 2025 Oct 30;16:1666974. doi: 10.3389/fpsyg.2025.1666974 (PMC12611650; doi:10.3389/fpsyg.2025.1666974)
Supplement: Supplementary file 1 [file Supplementary_file_1.docx]

**Appendix** A CL-AI-L2W Draft Scale

Instructions: The following questions ask about the amount of mental effort you invested in different activities while completing the AI-assisted writing task. Please rate your mental effort for each activity on the scale provided.

There are no right or wrong answers. We are interested in your personal experience.

Scale:

1 = Very, very low mental effort

2 = Very low mental effort

3 = Low mental effort

4. = Moderate mental effort

5 = High mental effort

6 = Very high mental effort

7 = Very, very high mental effort

Factor A: Planning & Organization (Traditional Writing Processes)

When completing the AI-assisted writing task, how much mental effort did it take you to...

1. Decide on the main argument or position for your essay.
2. Come up with initial supporting ideas and examples on your own (before using the AI).
3. Create a logical structure or outline for the entire essay.
4. Organize your arguments and evidence within each paragraph.
5. Ensure a smooth and logical flow between your paragraphs.
6. Decide what kind of information you needed to find or generate to support your argument.
7. Think about the overall introduction and conclusion of your essay.

Factor B: Prompt Engineering & Management (AI Interaction)

When completing the AI-assisted writing task, how much mental effort did it take you to...

1. Figure out the best way to phrase your initial questions (prompts) to the AI.
2. Think of specific keywords to guide the AI to give you the most useful response.
3. Rephrase or refine your prompts when the AI's first answer was not helpful.
4. Break down a complex task into a series of smaller, clearer prompts for the AI.
5. Ask the AI effective follow-up questions to get more details or different perspectives.
6. Manage the conversation with the AI to keep it focused on your writing goal.

Factor C: Critical Output Evaluation (AI Interaction)

When completing the AI-assisted writing task, how much mental effort did it take you to...

1. Judge whether the information provided by the AI was factually accurate.
2. Evaluate whether the AI's suggestions were relevant to your essay's topic and argument.
3. Assess the tone and style of the AI-generated text to see if it was appropriate for academic writing.
4. Decide which parts of the AI’s output to use and which parts to ignore or delete.
5. Check the AI’s text for potential bias or one-sided arguments.
6. Compare different responses from the AI to select the best one.
7. Identify and correct awkward or unnatural phrasing in the AI-generated text.

Factor D: Integration & Synthesis (AI Interaction & Writing)

When completing the AI-assisted writing task, how much mental effort did it take you to...

1. Paraphrase or rewrite AI-generated sentences to express them in your own words.
2. Blend the AI-generated text smoothly with your own writing.
3. Ensure that your personal authorial voice was not lost when using AI-generated content.
4. Connect your own ideas logically with the ideas or text provided by the AI.
5. Maintain a consistent grammatical and stylistic flow between your writing and the AI's text.
6. Synthesize information from multiple AI responses into a single, coherent paragraph.

Factor E: Language Expression & Revision (Traditional Writing Processes)

When completing the AI-assisted writing task, how much mental effort did it take you to...

1. Find the right vocabulary to express your specific ideas precisely.
2. Construct grammatically correct English sentences.
3. Focus on spelling and punctuation while writing and revising.
4. Revise the sentences you wrote yourself for clarity and correctness.
5. Review the entire essay to check for overall coherence and quality.
6. Ensure your final text directly answered the prompt and met all task requirements.
7. Manage your time effectively between writing, using the AI, and revising.
8. Concentrate on the writing task without getting distracted by the AI tool itself.
9. Monitor the overall logic and persuasiveness of your argument as it developed.

**Appendix** B: The Validated Cognitive Load Scale for AI-Assisted L2 Writing (CL-AI-L2W)

Instructions: The following questions ask about the amount of mental effort you invested in different activities while completing the AI-assisted writing task. Please rate your mental effort for each activity on the scale provided.

Scale: 1 = Very, very low mental effort | 2 = Very low | 3 = Low | 4 = Moderate | 5 = High | 6 = Very high | 7 = Very, very high mental effort

When completing the AI-assisted writing task, how much mental effort did it take you to...

Prompt Management

1. Figure out the best way to phrase your initial questions (prompts) to the AI.
2. Think of specific keywords to guide the AI to give you the most useful response.
3. Rephrase or refine your prompts when the AI's first answer was not helpful.
4. Break down a complex task into a series of smaller, clearer prompts for the AI.
5. Ask the AI effective follow-up questions to get more details or different perspectives.

Critical Evaluation

6. Judge whether the information provided by the AI was factually accurate.

7. Evaluate whether the AI's suggestions were relevant to your essay's topic and argument.

8. Assess the tone and style of the AI-generated text to see if it was appropriate for academic writing.

9. Decide which parts of the AI’s output to use and which parts to ignore or delete.

10. Check the AI’s text for potential bias or one-sided arguments.

Integrative Synthesis

11. Paraphrase or rewrite AI-generated sentences to express them in your own words.

12. Blend the AI-generated text smoothly with your own writing.

13. Ensure that your personal authorial voice was not lost when using AI-generated content.

14. Connect your own ideas logically with the ideas or text provided by the AI.

Authorial Core Processing

15. Decide on the main argument or position for your essay.

16. Create a logical structure or outline for the entire essay.

17. Find the right vocabulary to express your specific ideas precisely.

18. Construct grammatically correct English sentences.

**Appendix** C: Item Reduction Process

Table C1. Rationale for Item Deletion to Final Scale (18 items)

| Phase 2: Item Analysis (35 → 30 items) |  |  | Corrected Item-Total Correlation < .40 |
| --- | --- | --- | --- |
|  | 13 | Manage the conversation with the AI. | r = .39 |
|  | 5 | Ensure a smooth and logical flow between paragraphs. | r = .38 |
|  | 29 | Focus on spelling and punctuation. | r = .35 |
|  | 33 | Manage your time effectively. | r = .34 |
|  | 34 | Concentrate on the writing task without getting distracted. | r = .31 |
| Phase 3: EFA Refinement (30 → 18 items) |  |  | Low Loading or Significant Cross-Loading |
|  | 20 | Identify awkward phrasing in AI text. | Cross-loading on F2 (CE, .51) and F4 (ACP, .43). |
|  | 30 | Revise sentences you wrote yourself. | Cross-loading on F3 (IS, .44) and F4 (ACP, .49). |
|  | 31 | Review entire essay for overall coherence. | Weak primary loading (.46) and cross-loadings. |
|  | 4 | Organize arguments within each paragraph. | Redundant with Item 3; weaker loading (.42). |
|  | 25 | Maintain consistent flow between your text and AI's. | Redundant with Item 22; weaker loading (.53). |
|  | 32 | Ensure final text answered the prompt. | Conceptually closer to F2 (CE); weaker loading (.41). |
|  | 35 | Monitor overall logic of your argument. | Redundant with Item 1; weaker loading (.48). |
|  | 2 | Come up with initial ideas on your own. | Primary loading < .40 (.35). |
|  | 6 | Decide what info you needed to find/generate. | Primary loading < .40 (.37). |
|  | 7 | Think about the intro and conclusion. | Primary loading < .40 (.38). |
|  | 19 | Compare different responses from the AI. | Primary loading < .40 (.31) and conceptual ambiguity. |
|  | 26 | Synthesize info from multiple AI responses. | Primary loading < .40 (.39). |

**Appendix** D: Detailed Exploratory Factor Analysis Results

Table D1. Pattern Matrix and Communalities for the Final 18-Item CL-AI-L2W Scale

| **Item No.** | **Item Content (Summary)** | F1 (PM) | F2 (CE) | F3 (IS) | F4 (ACP) | Communalities (h²) |
| --- | --- | --- | --- | --- | --- | --- |
| Prompt Management (PM) |  |  |  |  |  |  |
| 10 | Rephrase/refine prompts when AI's answer was not helpful. | **.871** | .108 | .093 | .031 | .779 |
| 8 | Figure out the best way to phrase initial questions. | **.852** | .145 | .120 | .079 | .763 |
| 12 | Ask effective follow-up questions. | **.810** | .179 | .101 | .054 | .726 |
| 9 | Think of specific keywords to guide the AI. | **.793** | .131 | .084 | .105 | .688 |
| 11 | Break down a complex task into smaller prompts. | **.754** | .090 | .155 | .139 | .657 |
| Critical Evaluation (CE) |  |  |  |  |  |  |
| 15 | Evaluate if AI suggestions were relevant to your argument. | .122 | **.863** | .138 | .068 | .789 |
| 17 | Decide which parts of AI output to use and which to ignore. | .101 | **.830** | .205 | .099 | .754 |
| 14 | Judge if AI information was factually accurate. | .079 | **.811** | .112 | .051 | .703 |
| 16 | Assess the tone and style of the AI text. | .138 | **.772** | .187 | .127 | .701 |
| 18 | Check the AI text for potential bias. | .061 | **.724** | .093 | .080 | .598 |
| Integrative Synthesis (IS) |  |  |  |  |  |  |
| 22 | Blend AI text smoothly with your own writing. | .114 | .175 | **.851** | .149 | .781 |
| 21 | Paraphrase or rewrite AI sentences in your own words. | .092 | .153 | **.822** | .121 | .723 |
| 24 | Connect your own ideas logically with AI ideas. | .129 | .198 | **.804** | .185 | .745 |
| 23 | Ensure your personal authorial voice was not lost. | .071 | .120 | **.743** | .245 | .658 |
| Authorial Core Processing (ACP) |  |  |  |  |  |  |
| 3 | Create a logical structure or outline for the essay. | .088 | .103 | .168 | **.840** | .762 |
| 1 | Decide on the main argument or position for your essay. | .109 | .081 | .111 | **.802** | .691 |
| 28 | Construct grammatically correct English sentences. | .054 | .063 | .141 | **.731** | .588 |
| 27 | Find the right vocabulary to express your ideas precisely. | .081 | .095 | .123 | **.690** | .521 |

Note. Extraction Method: Principal Axis Factoring. Rotation Method: Oblimin with Kaiser Normalization. Factor loadings > .40 are in bold. Item numbers correspond to the draft scale in Appendix A.

**Appendix** E: Descriptive Statistics for the Final 18 Scale Items

Table E1. Item-Level Descriptive Statistics (N = 305)

| Item No. | Item Content | Factor | M | SD | Skewness | Kurtosis |
| --- | --- | --- | --- | --- | --- | --- |
| PM1 | Figure out the best way to phrase your initial questions (prompts) to the AI. | PM | 4.61 | 1.55 | -0.45 | -0.61 |
| PM2 | Think of specific keywords to guide the AI to give you the most useful response. | PM | 4.70 | 1.61 | -0.51 | -0.70 |
| PM3 | Rephrase or refine your prompts when the AI's first answer was not helpful. | PM | 4.82 | 1.58 | -0.60 | -0.55 |
| PM4 | Break down a complex task into a series of smaller, clearer prompts for the AI. | PM | 4.33 | 1.70 | -0.28 | -0.88 |
| PM5 | Ask the AI effective follow-up questions to get more details or different perspectives. | PM | 4.29 | 1.65 | -0.21 | -0.81 |
| CE1 | Judge whether the information provided by the AI was factually accurate. | CE | 4.95 | 1.60 | -0.75 | -0.40 |
| CE2 | Evaluate whether the AI's suggestions were relevant to your essay's topic and argument. | CE | 5.01 | 1.54 | -0.81 | -0.25 |
| CE3 | Assess the tone and style of the AI-generated text to see if it was appropriate for academic writing. | CE | 4.75 | 1.62 | -0.55 | -0.73 |
| CE4 | Decide which parts of the AI’s output to use and which parts to ignore or delete. | CE | 4.88 | 1.59 | -0.68 | -0.48 |
| CE5 | Check the AI’s text for potential bias or one-sided arguments. | CE | 4.45 | 1.72 | -0.33 | -0.95 |
| IS1 | Paraphrase or rewrite AI-generated sentences to express them in your own words. | IS | 4.65 | 1.68 | -0.48 | -0.77 |
| IS2 | Blend the AI-generated text smoothly with your own writing. | IS | 4.50 | 1.65 | -0.39 | -0.82 |
| IS3 | Ensure that your personal authorial voice was not lost when using AI-generated content. | IS | 4.30 | 1.75 | -0.25 | -1.01 |
| IS4 | Connect your own ideas logically with the ideas or text provided by the AI. | IS | 4.15 | 1.71 | -0.18 | -0.98 |
| ACP1 | Decide on the main argument or position for your essay. | ACP | 3.60 | 1.50 | -0.11 | -0.85 |
| ACP2 | Create a logical structure or outline for the entire essay. | ACP | 3.75 | 1.55 | -0.15 | -0.90 |
| ACP3 | Find the right vocabulary to express your specific ideas precisely. | ACP | 3.30 | 1.60 | 0.10 | -0.80 |
| ACP4 | Construct grammatically correct English sentences. | ACP | 3.27 | 1.58 | 0.12 | -0.75 |

Note. N = 305. The scale for all items was a 7-point Likert scale (1 = Very, very low mental effort to 7 = Very, very high mental effort). Item numbers (PM1-ACP4) correspond to the final scale items in Appendix B.
